# Cost-efficacy analysis of the MONET trial using Spanish antiretroviral drug prices

**DOI:** 10.1186/1758-2652-13-S4-P235

**Published:** 2010-11-08

**Authors:** JR Arribas, A Hill, I Garcia, A Anceau

**Affiliations:** 1 Hospital La Paz, Madrid, Spain;; 2 Liverpool University, Liverpool, UK; 3 Janssen-Cilag, Madrid, Spain;; 4 Janssen France, Issy-les-Moulineaux, France

## Background

In virologically suppressed patients, switching to DRV/r monotherapy maintains HIV RNA suppression, and could also lower treatment costs.

## Methods

In the MONET trial 256 patients with HIV RNA <50 copies/mL on current HAART for over 24 weeks (NNRTI based (43%), or PI based (57%)), switched to DRV/r 800/100 mg once daily, either as monotherapy (n=127) or with 2NRTI (n=129). The Spanish costs per patient with HIV RNA below 50 copies/mL were calculated, using a "switch included" analysis at Week 96, to account for additional antiretrovirals taken after initial treatment failure. Published prices were used.

## Results

In the ITT switch included analysis, HIV RNA <50 copies/mL by Week 96 was 92.1% versus 90.7% in the DRV/r monotherapy and control arms. No patients in either arm developed phenotypic resistance to DRV. Before the trial, the mean annual cost of antiretrovirals was €4612 for patients on NNRTI based HAART, and €9217 for patients on PI based HAART. During the MONET trial, the mean annual per-patient cost of antiretrovirals was €9915 in the triple therapy arm, of which 45% was from NRTIs and 55% from PIs. The mean per-patient cost in the monotherapy arm was €5915, a saving of 40%. We estimated 65,000 people treated with antiretrovirals in Spain (50% NNRTI based, 50% PI based) and 15% of patients (9,750) eligible for PI monotherapy. A switch to DRV/r monotherapy could cut the two-year cost of antiretroviral treatment for these patients, from €137 million to €115 million, a saving of €22 million over two years.

## Conclusions

Based on the MONET results, the lower cost of DRV/r monotherapy versus triple therapy in Spain would allow more patients to be treated for a fixed budget, or a saving of up to €22 million over two years, if all eligible patients were switched, while maintaining HIV RNA suppression below 50 copies/mL.

